# Mycelium-Based Composites Using Minimally Processed Industrial Hemp Biomass: Impact of Species and Feedstock Ratio on Mechanical Performance Compared to Polystyrene Packaging

**DOI:** 10.3390/polym18030400

**Published:** 2026-02-03

**Authors:** Radika Bhaskar, Tanisha Rutledge, Kevin Trangone, Oneal Latimore

**Affiliations:** 1 Department of Engineering, Thomas Jefferson University, 4201 Henry Avenue, Philadelphia, PA 19144, USA; 2 Eastern Hemp Company, Philadelphia, PA 19144, USA

**Keywords:** industrial hemp, mycelium, biodegradable, alternative to styrofoam, green packaging

## Abstract

Mycelium-based composites (MBCs\) are formed from lignocellulosic substrates and biopolymer matrices derived from fungal mycelium. Due to their low fossil energy demand and biodegradability, MBCs represent a versatile and sustainable material suitable for a range of applications, with increasing interest focused on packaging. Hemp fibers are an example of natural fibers with great promise as a substrate to improve the mechanical properties of MBCs. However, the separation of bast and hurd fiber requires processing and commercial-scale facilities that are logistically challenging and may be cost-prohibitive. Here, the potential for minimally processed hemp, with no separation of fibers, is evaluated for the first time to demonstrate feasibility as a substrate for MBCs. Screening included different fiber ratios combined with three different, locally available mushroom strains, which are among the most common in MBC research. The resulting MBCs were tested as an alternative to environmentally harmful expanded polystyrene (EPS, or polystyrene foam), with a focus on compressive strength to reflect load-bearing performance. Some MBCs revealed mechanical performance that met or exceeded EPS, demonstrating the utility of minimally processed hemp fiber in biocomposites for safer packaging.

## 1. Introduction

Expanded polystyrene (EPS; e.g., Styrofoam or polystyrene foams) is widely used for packaging due to beneficial properties including low cost, low weight, shock absorption, and insulation [1]. However, the environmental impacts of its production and disposal are severe. Polystyrene is a petrochemical derivative, and the manufacturing of EPS has a high global warming potential due to the resources and energy required [2,3]. EPS end-of-life is also of concern as it is neither biodegradable nor compostable [3]. While the material is safely recyclable, few areas implement EPS recycling, resulting in most products ending up in landfills, leading to further greenhouse gas emissions [4]. Thus, taking the entire life-cycle perspective, the product has a high carbon footprint [1].

As businesses shift away from EPS, there is an enormous opportunity for sustainable alternatives. Materials derived from mycelium, the root structure of mushrooms, have many advantages, including 1. low energy requirements, 2. low carbon footprint, 3. The ability to meet many structural or functional needs [5], and 4. biodegradability [6]. These benefits have spurred a growing trend of using fungi as the source for bio-based materials [7].

Mycelium-based composites (MBCs) combine the mycelium, which includes the biopolymers chitin and glucan as the matrix or ‘natural glue’, plus a lignocellulosic fiber as substrate [8]. Fungal species from within the ‘white rot’ mode of wood decomposition can produce enzymes that permit the breakdown of lignin [5], resulting in a higher percentage of cellulose and thereby creating materials with favorable mechanical properties. Within the white rot group, there exists substantial biological variation that can influence the density and compressive strength of the end product [5,9], thus screening a variety of species is critical.

The selection of substrate is also consequential to the final product characteristics. The choice of natural fiber can influence the material’s physical, mechanical, and thermal properties [10]. Hemp fibers are an example of natural fibers with great promise to improve the mechanical properties of the bio-composite and are particularly attractive as a substrate for mycelium matrices due to their high cellulose content and lower lignin content [11].

In addition to considerations of material performance, the selection of substrate presents an opportunity to strategically incorporate lignocellulosic materials from regional organic waste streams. Incorporation of residues from agriculture can provide value to underutilized biomass feedstocks and advance a circular regional bioeconomy [12]. This is the case for industrial hemp (*Cannabis sativa* L.). In the US, over 45,000 acres were planted in 2024, representing a 64% increase from the previous year, with production anticipated to increase [13]. Analyses have shown that when grown for CBD extracted from the hemp flowers, about 90% of the biomass ends up as residual waste [14]. Harvested industrial hemp stalks can be pre-treated and processed to separate the outer hemp fibers used for textiles, among other high-value applications, and the inner hemp hurd for products such as hempcrete [15]. The hemp hurd has also been successfully used as a substrate for numerous mycelium species [5]. However, the processing of hemp stalks, including chemical pre-treatment and mechanical separation into bast (long fiber) and hurd (also called shives), requires specialized equipment [16], and the time-intensive methods and machinery involved in fiber extraction impact the resulting potential value of the derived products [17]. Even simpler, more environmentally friendly methods of fiber separation still require chemical pre-treatment followed by physical milling [18]. As a result, the minimally processed harvested hemp stalks from local industrial hemp farmers require investigation to determine whether there are higher-value products that can be developed without such involved processing.

While the importance of the choice of feedstock and selection of mycelial strain has been documented, there is less systematic study of how the ratio of substrate to inoculum may influence physical and mechanical properties. There is some suggestion that increasing the percentage of the hemp substrate can increase the compressive strength of the resulting biocomposite [19]. Therefore, we also evaluate varying inoculum/feedstock ratios to assess whether a stronger material could be made by incorporating more of the feedstock, thus providing a means to utilize even more of the currently low-value organic waste material.

Our work aims to explore developing an MBC derived from underutilized hemp stalks from industries in Pennsylvania. By harnessing regional agricultural biomass, we can develop a cost-effective, locally sourced alternative to plastic packaging. We leverage local supply chains to achieve the overarching goal of developing sustainable packaging, an aim that is well-aligned with sustainability trends observed globally. Our objectives are to evaluate the feasibility of using minimally processed hemp stalks as a substrate by screening three locally available fungal species and two different inoculum/feedstock ratios in a full-factorial experiment. We characterize the resulting MBCs for physical and mechanical properties to assess if they are comparable to EPS.

## 2. Materials and Methods

### 2.1. Materials

#### 2.1.1. Fungal Species

We selected three mushroom species that are widely available and have important characteristics related to the formation of composites [20]. For example, all are white-rot fungi, a functional category referring to species generally within the Basidiomycota that have enzymes that allow for lignin degradation [21]. Some strains may preferentially consume lignin without impacting cellulose, thus improving MBC strength [5], though interspecific differences exist among white-rot fungi in lignin degradation [22].

The selected fungal strains (*Ganoderma sessile*, *Trametes versicolor*, and *Pleurotus ostreatus*) are species from the top three most studied genera for MBC production [23] and have been shown to grow on a range of lignocellulosic materials [5]. *Ganoderma* species have been found to have high growth rates and are already used in MBC products on the market [24], which makes them a promising candidate for study. The selected species also vary in how their mycelium networks are structured. *P. ostreatus* has a monomitic hyphal system, meaning it has only one form of hyphae (generative), whereas *T. versicolor* and *G. sessile* are trimitic (with generative, skeletal, and binding hyphae) [25,26]. Species with trimitic systems may exhibit greater structural integrity, resulting in improved mechanical properties compared to monomitic species [26]. Collectively, the three species present a range of attributes relevant to assessing MBC performance.

Mushroom spawn consisting of fungal culture of each strain of mycelium inoculated in the cereal grain millet was procured from MycoSymbiotics (Lemoyne, PA, USA) and served as the inoculum for all trials.

#### 2.1.2. Minimally Processed Chopped Hemp

**Figure 1:**
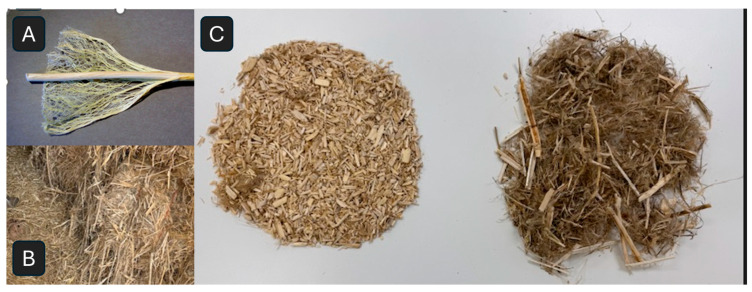
Photos depicting industrial hemp fibers, including (**A**) hemp stalks comprising both bast and hurd, (**B**) bales of air-dried harvested stalks with no processing or separation of fiber types, and (**C**) a comparison of processed, separated (decorticated) hemp hurd on the left versus minimally processed chopped hemp used in this study on the right. Chopped hemp is a mix of fine, longer outer fiber and coarser, shorter hurd fibers.

Stalks of industrial hemp (*Cannabis sativa* L.) comprise the outer longer fibers as well as the inner hurd (Figure 1A). In this study, minimally processed hemp stalk material was provided by Hemp Alternative (Chester County, PA, USA). The hemp stalks that had been harvested and air-dried in bales (Figure 1B) were subsequently chopped in a grain mill, resulting in a mix of the finer outer fibers and the coarser inner hurd fibers, referred to as chopped hemp. As seen visibly in comparing the materials (Figure 1C), the chopped form shown on the right differs from hemp hurd predominantly due to the inclusion of the longer outer fibers as well as the larger pieces of coarse hurd.

To put the material properties of minimally processed chopped hemp into context, we compared it to building-grade quality hemp hurd available on the market, with European certification to ensure consistency in granule size and other characteristics (visible comparison in Figure 1C). Hemp hurd is the most common form of hemp used in MBCs [27,28]. A comparison of the distribution of different size categories is shown in Table 1 after 50 g of each sample is separated by sieving into different size categories, following a similar protocol to [29], and weight percentages are calculated for each size.

Chopped hemp has a higher percentage of larger-sized components, largely made up of the longer outer fibers.

Water holding capacity was measured by soaking the hemp substrates in water for 5 min, then straining the material without squeezing until water droplets ceased forming, to ensure field capacity. Five trials were performed for 300 g of chopped hemp compared to hemp hurd to calculate the percentage of water absorbed. Chopped hemp had a field capacity of 76%, while hemp hurd had a field capacity of 68%, and chopped hemp also had a lower bulk density (0.11 g/cm^3^) compared to hemp hurd (0.15 g/cm^3^). Overall, chopped hemp with the mix of hurd and outer fibers results in a substrate of lower density, higher field capacity, and a different size distribution compared to hemp hurd.

### 2.2. Fabrication and Feedstock Ratios

The fabrication process generally followed methods used by other studies (see e.g., [30]). In brief, the substrate was steam-sterilized through autoclaving fully water-saturated hemp materials for 30 min at 121 °C following typical procedures [5], and then an aseptic technique was used to prevent contamination for all subsequent steps. The mushroom spawn was mixed with the sterilized hemp following an inoculum/substrate volumetric ratio of either 1:2 or 1:4, and the resulting mass was recorded along with the volume of water necessary to reach field capacity (Table 2).

**Figure 2:**
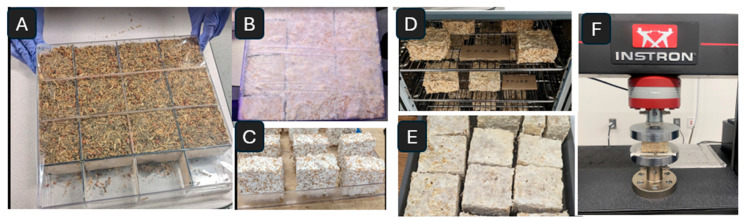
(**A**) Inoculum-hemp substrate mix packed into plastic molds with square inserts to create 75 mm × 75 mm specimens. (**B**) Materials covered in plastic and placed in grow tents until (**C**) all specimens have demonstrable white mycelium covering all sides. (**D**) Samples oven-dried for 24–36 h at 82 °C. (**E**) Dried specimens used for characterization and testing, including (**F**) the Instron testing following ASTM D1621.

The hand-mixed materials were then packed into clear plastic molds and pressed with an even 25-pound weight to distribute the substrate as uniformly as possible. Materials were grown in 75 mm × 75 mm square-shaped molds to meet ASTM testing criteria (see Figure 2), packed to a height of 40 mm, and placed in plastic bags in grow tents with humidity levels of 65% for 14 days until fully myceliated (with white mycelial growth evenly visible on all sides). We observed that *P. ostreatus* and *G. sessile* samples were visibly mycelated earlier; however, *T. versicolor* samples had an uneven and weaker growth. Therefore, we left all samples for the same 14-day duration.

Samples were removed from molds and oven-dried at 82 °C for the minimum number of hours needed to deactivate the mycelium through desiccation. This was determined by weighing samples after oven-drying until no change in mass was detected. For *P. ostreatus* and *G. sessile*, this required 24 h, and for *T. versicolor*, 36 h to deactivate the mycelium.

### 2.3. Characterization

#### 2.3.1. MBC Density

The dry density of the samples was measured prior to mechanical testing. Sample dimensions were measured using digital calipers, and each sample’s mass was measured on a Sartorius laboratory balance accurate to 0.01 g. Dry density was calculated using Equation (1):

*d* (kg/m^3^) = *m*/*v* (1)

where *d* = dry density (kg/m^3^); *m* = the mass of each sample after oven-drying (kg), and *v* = the volume of each sample after oven-drying (m^3^).

#### 2.3.2. Mechanical Testing

Compressive properties, specifically compressive strength and Young’s modulus, were measured according to the Standard Test Method for Compressive Properties of Rigid Cellular Plastics (ASTM D1621, [31]). Compressive strength, σ, was chosen as an important indicator of mechanical strength, as it reflects the ability to resist crushing and withstand evenly distributed loads, as might occur when used for food packaging. Young’s modulus, E_c_, is a measure of a material’s compressive stiffness. It is measured in the linear elastic region of a load–displacement curve, in which an object can recover to its original shape once the load is removed and does not display permanent deformation.

All samples were conditioned for a minimum of 24 h in controlled ambient conditions (65 ± 5% RH and 21 ± 2 °C) and then tested using an Instron universal test instrument (Instron Corp., Canton, Mass.) equipped with a 100 kN load cell. The crosshead rate was set to 10% of the specimen thickness per minute, on average 4 mm/min, and stopped once samples had reached 50% strain.

The calculation of compressive strength is shown in Equation (2). As per the test method, the stress used in the calculation should be the stress at the yield point, provided it occurs before 10% deformation; if no yield point is observed, the stress at 10% strain is used instead.

*σ* (kPa) = *F*/*A* (2)

where *σ* = compressive stress (kPa); *F* = compressive load (N); and *A* = initial cross-sectional area of the specimen (mm^2^).

The calculation of modulus, *E_c_*, is shown in Equation (3).

*E_c_* = *WH*/*AD* (3)

where *W* = load (N); *H* = initial specimen height (mm); *A* = cross-sectional area (mm^2^); and *D* = deformation or strain (mm).

### 2.4. EPS Benchmark Testing

To compare the performance of the various MBCs to EPS, we cut specimens from Styrofoam coolers (polystyrene foam insulating coolers; commonly available brands, including Polar Tech) using a hot wire to characterize and test, following the above density and mechanical testing methods, while conducting MBC testing.

### 2.5. Statistical Analysis

Ten replicate samples of MBCs were used for each combination of species and ratio, which is a higher replication than is often used for testing. Two-way analysis of variance (ANOVA) was used to evaluate the effects of the independent variables (species and ratio) on sample density, compressive strength, and compressive modulus, analyzed using Python 3.1.2, particularly the *statsmodels* package. Statistical significance of the independent variables and their interaction was assessed at *p* < 0.05. ANOVA output is presented in the Supplementary Material.

## 3. Results and Discussion

### 3.1. Density

MBC samples varied in density from 155 to 270 kg/m^3^, with an average overall density of 207.5 kg/m^3^. These values fall within the large range of densities that have been reported for MBCs [32]. MBCs with hemp hurd-based substrates have generally been reported to have lower composite dry density compared to other substrates [27,33], though direct comparisons are difficult because substrate processing and fiber size, as shown in that study, as well as substrate-to-spawn ratio, as demonstrated in this one, both influence MBC density. These findings add to the body of work that suggests substrate choice and processing can greatly modify MBC density. The variability of MBC density and the importance of substrate are observed repeatedly across a number of different species (e.g., [20,27,34]), with substrate carbon/nitrogen ratios and pH noted as important factors determining mycelial growth rates and density for the three genera in this study [35].

**Figure 3:**
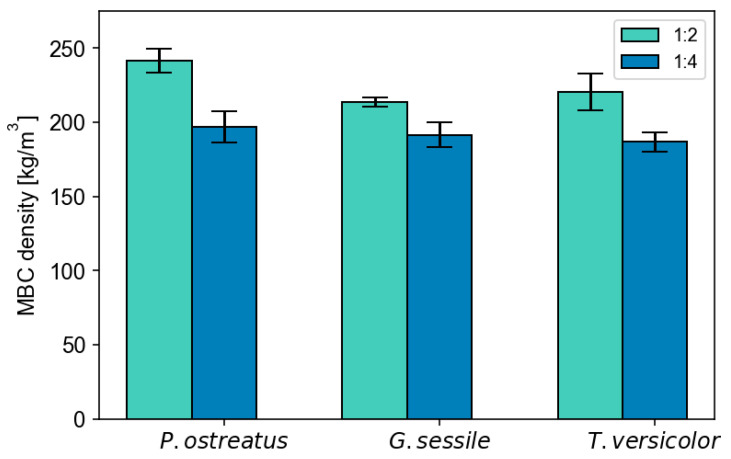
MBC density average (± standard error) for *P. ostreatus*, *G. sessile*, and *T. versicolor* species, for each ratio.

Based on the two-way ANOVA, ratio was a significant factor influencing MBC density (F = 23.9, *p* < 0.001), with a 1:2 ratio exhibiting significantly higher density, as shown in Figure 3 (also see Supplementary Table S1 and Figure S1). Species was not a significant factor (*p* > 0.05), and neither was the interaction of species × ratio (*p* > 0.05). This overall suggests that all screened fungal species had a similar response, resulting in a consistently lower MBC density at the higher inoculum/substrate ratio.

The 1:4 ratio results from an increase in hemp substrate relative to millet-based spawn, which was the inoculum. Because specimen density is largely influenced by substrate, the changes in density may be due to the greater density of myceliated millet with less void space compared to the chopped hemp. Grains have been found in other MBCs to increase the overall composite density [36], which is consistent with the observed increase in density in the substrates with a greater percentage of millet. Altering the type of grain for initial inoculation could be a means to modify MBC density, in addition to varying the amount of substrate included; further, as grain inoculum has been found to have higher density than liquid inoculum, switching to a liquid inoculum could be an option to reduce overall MBC density [37].

### 3.2. Mechanical Behavior

#### 3.2.1. Compressive Strength

Compressive strength is an important property for materials that serve as packaging [38], and MBCs have been widely seen as a promising alternative to EPS packaging [39,40]. To narrow the range of variables that influence composite properties, here we deliberately evaluate a factorial experiment that allows us to test for the influence of fungal strain, substrate ratio, and the interaction.

**Figure 4:**
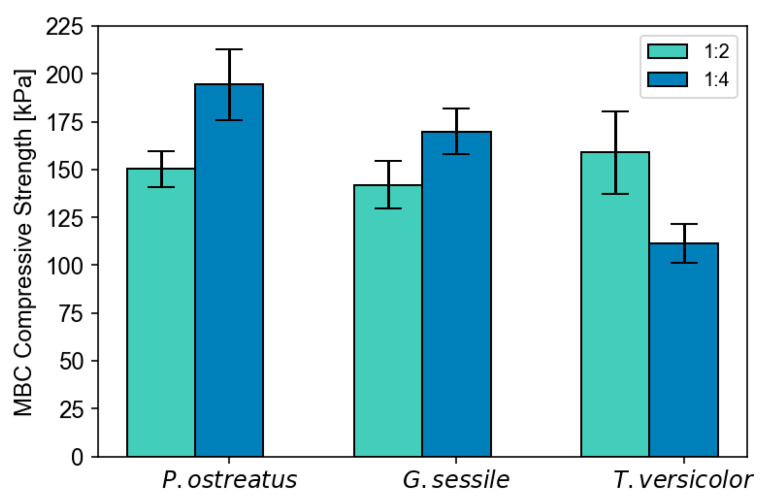
Average ± standard error compressive strength at 10% deformation for fungal species × ratio MBC.

Per the ASTM test method, here we report the compressive strength at 10% deformation, as no yield point occurred prior to 10% deformation. As seen in Figure 4, generally fungal strains formed composites of comparable compressive strength with wide variation (CV% ranging from 20 to 35). A two-way ANOVA analysis revealed significant differences among species influenced by the level of substrate ratio. Species displayed differential responses to an increasing ratio of inoculum/chopped hemp, resulting in a significant interaction term (F = 5.7, *p* < 0.01) in the statistical model (Table S1, Figure S1). While *P. ostreatus* and, to some extent, *G. sessile* have increased compressive strength at the 1:4 ratio, *T. versicolor* decreases in compressive strength. These differences could, in part, relate to differential species-specific preferences for the substrate. Another study examining *T. versicolor* growth on varied substrates, including hemp shives, found comparable values on average, and a wide range of compressive strengths from 29 to 225 kPa [29].

Overall, we observed an average MBC compressive strength of 154.6 ± 48.9 kPa, which is within the range reported for MBCs (see, for example, [36,41]). However, drawing direct comparisons to other results is challenging. Even when comparing the same fungal strain and the same or similar substrate, the lack of standard methodologies for the production of this material, combined with variation in testing methodologies for the same properties, can contribute to wide differences in findings and hinder the ability to draw generalizations. Here, for example, compressive strength is reported at 10% deformation following the ASTM D1621 method conventions, which is a test method technically equivalent to the ISO 844 method [31]; both methods are for rigid cellular plastics. However, MBCs are tested for compression resistance using a wide variety of methods [42], including for wood-based structures, some of which report the maximum force or compressive strength at higher percent deformation.

To illustrate the variation in compressive strength, Table 3 provides a comparison of compressive strength reported at deformation between 10 and 35%, as indicated, with mycelium biocomposites fabricated using the same fungal genus or exact species used in this study and grown with some form of hemp as the substrate.

Reviewing the findings, it is challenging to draw generalizations when there is no consistency in the percentage of reported strains. For example, a reported value for the same species grown on the same substrate can be nearly four times higher at 35% deformation versus 10% deformation [27], observed for both *P. ostreatus* and *G. lucidum* as shown in Table 3. Thus, trying to make recommendations for design parameters to suit specific applications (e.g., [47]) requires factoring in this additional dimension of information.

Recognizing the wide diversity of species responses to the same substrates and conditions is also important. This knowledge gap can be addressed by further studies that include multiple species in the overall experimental design. Species may demonstrate contrasting responses or varied emergent composite properties. Another study also observed species-by-substrate interactions, with compressive strength increasing for one species and decreasing for another on two substrates [45].

Table 3 also highlights that the range of compressive strengths in this study is towards the higher end of what has been observed for similar or identical species growing on hemp-based substrates, particularly noting that some of the findings present compressive strength at higher percentage deformation than this study. The compressive strength exceeds even what has been reported for Ecovative [45], which is on the market as a packaging material using hemp hurd. This collectively provides strong evidence that minimally processed hemp can be a viable substrate for mycelium-based composites and points to novel uses for a currently underutilized residue in agriculture.

#### 3.2.2. Compressive Modulus of Elasticity (Young’s Modulus)

Compressive modulus of elasticity (also called Young’s modulus) reflects the stiffness of the material in the elastic (reversible) phase, with a higher stiffness reflecting a material’s greater resistance to deformation. We found the compressive modulus of elasticity significantly differed between species, which was further moderated by the hemp ratio, resulting in a significant interaction (F = 6.4, *p* < 0.01) (Table S1). For both *P. ostreatus* and *G. lucidum*, higher ratios had higher stiffness; the reverse was true for *T. versicolor*.

MBCs based on hemp hurd substrates have been found to have higher stiffness compared to other lignocellulosic materials, including flax and sawdust [33]. As seen in Table 3 comparing studies of hemp hurd, there is variation in reported elastic modulus, and the values observed in this study are comparable and on the higher end of the range, further speaking to the promise of minimally processed hemp stalks as a substrate for MBCs. Interestingly, processing of lignocellulosic substrate to reduce particle size had a larger impact than substrate type [33], which suggests that any process of size reduction in the minimally processed hemp could increase material stiffness.

#### 3.2.3. Elastic Modulus and Specimen Density

It has been hypothesized that mycelium biocomposites behave in the same way as plastic foam, with a similar range of values and a positive relationship between elastic modulus and density when compared in an Ashby chart [48]. This positive correlation has been observed experimentally [49]; this relationship may become evident only over a larger range in biocomposite densities, such as can be achieved by pressing samples into more rigid materials during the processing stage [50].

**Figure 5:**
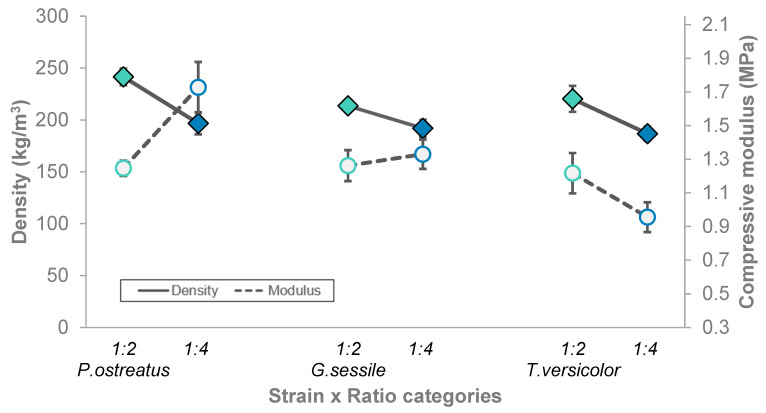
Comparison of MBC specimen density and compressive Young’s modulus for each fungal species and each inoculum/substrate ratio (colors as in Figure 3 and Figure 4 for ratio). Filled diamond and solid line = MBC density (kg/m^3^), and unfilled circle and dashed line = compressive modulus.

In this study, shifts in MBC density are not always paralleled by shifts in elastic modulus (Figure 5). While *T. versicolor* demonstrates an increase in stiffness with an increase in density, the other two species do not demonstrate this expected correlation, and in fact, *P. ostreatus* provides a contrasting pattern. This inconsistent relationship is observed in other studies as well [27,32]. Therefore, the relationships among specimen density, elastic modulus, and compressive strength are not in line with expectations for EPS foams, as discussed below in more detail.

### 3.3. Comparison with EPS

The density of EPS may range widely based on application, but most commonly for protective food packaging, it is designed to be lightweight and is 96+ percent air [51], with densities ranging from 10 to 25 kg/m^3^ [52]. In this study, polystyrene foam cut from a packaging cooler had a density that fell in this range and was comparable to what other studies have found [29]; see Table 4. In comparison, MBC density in this study was nearly an order of magnitude higher.

Relatively high MBC density is typical, as observed in numerous design factors [27,37]. Though density reduction is needed to reach values comparable with EPS, it may not prohibit adoption for the market. For example, Ecovative materials are already in use despite higher density, and current green biodegradable products on the market have even higher densities [27], suggesting MBCs may still be viable in this application even with elevated values compared to EPS.

The behavior of expanded polystyrene in response to compressive stress has been widely studied both through modeling of closed-cell foam as well as through empirical study. Many of the mechanical properties of interest, including compressive stress at low levels of strain and the compressive elastic modulus, are positively correlated with material density [53,54]. This can be observed in Table 4, which compiles values from the literature [38,51] as well as from this study for EPS density, compressive strength (at 10% strain or as indicated), and elastic modulus. Evaluations of EPS for packaging find that a higher-density specimen results in a higher compressive strength and a higher elastic modulus (Table 4).

**Table 4:** Physical and mechanical characteristics of EPS from the literature review, as well as ASTM Standard Specification [31], and this study.

Density (kg/m^3^)	Compressive Strength (kPa)	%Strain	Elastic Modulus (MPa)	Ref.
11	53.38	10		[51]
15	70.55	10		[51]
20	111.9	10		[51]
25	138.14	10		[51]
22	165	10	4.7	[38]
23	177	10	5.2	[38]
16.3 ± 0.9	86.2 ± 7.3	15	3.26 ± 0.4	[55]
26.8 ± 0.6	171 ± 4.9	15	7.35 ± 0.2	[55]
22	104	10		[31]
21.5 ± 0.4	140.6 ± 2.7	10	5.9 ± 0.7	This study

In contrast, the relationships among MBC density, elastic modulus, and compressive strength are not necessarily correlated [32,56] or generalizable, which was also the case in this study.

Overall, MBC specimens exhibit values of compressive strength that meet or largely exceed the EPS compressive strength values that we measured and that are found in the literature (Table 4), including the minimum value listed as the ASTM Standard Specification for EPS of 104 kPa at 10% deformation [31]. This was true for at least some combination with all three species, providing first-of-its-kind evidence that composites made with minimally processed hemp have the potential to meet EPS compressive strength performance.

*P. ostreatus* has one of the highest values, particularly when grown with a higher proportion of hemp substrate, and further testing is warranted to see if material properties can be improved with decreasing particle size and increasing hemp ratio. This may also help to increase the elastic modulus, which for all MBC values was lower than EPS, suggesting a lower material stiffness.

## 4. Conclusions

Here we evaluate a novel MBC with minimally processed hemp stalks as substrate and demonstrate that it compares favorably to other MBCs made with hemp substrates requiring more energy- and machinery-intensive processing. Furthermore, the compressive strength meets or exceeds that of EPS-based packaging. These results are timely, as across the globe, legislation banning EPS is creating momentum to develop alternative biopolymer-based sustainable options [57] and advance a circular economy framework [58].

Industrial hemp is a sustainable crop, requiring little water and chemical application [59]. Minimally processed industrial hemp stalks represent readily available and currently underutilized agricultural by-products. Particularly in light of the competitive low-cost production of MBCs [39], there is a growing trend of commercialization [60]. Consequently, these findings identify an opportunity, particularly with the minimal processing of this material, to create a cost-effective, high-value composite with a packaging application. Future directions can evaluate further size reduction in chopped hemp to improve material stiffness and/or density.

**Figure 6:**
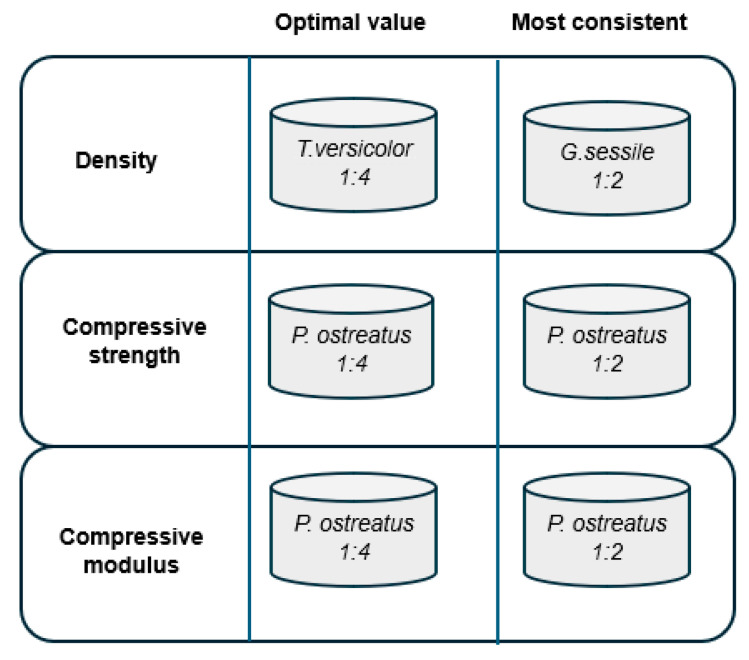
Comprehensive conceptual summary. ‘Optimal value’ identifies the particular MBC species x ratio combination with an average value to match the desired attribute state, for example, the lowest MBC density, highest compressive strength, and highest compressive modulus. ‘Most consistent‘ depicts the particular species × ratio combination with the lowest standard deviation for that attribute, reflecting the lowest variability among replicates.

We also highlight the importance of considering the interaction of substrate with a variety of fungal strains, as they interact differentially and provide a variety of ways to optimize design and MBC performance. As shown in Figure 6, the suitability of a particular combination of species x ratio will depend on the desired outcome, whether a particular average characteristic is preferred (‘optimal value’) or whether lower variability is prioritized (‘most consistent’).

This form of synthesizing the findings elucidates a potential contrasting influence of ratio, as the higher ratio (substrates incorporating more hemp) may result in MBCs with the desired characteristics on average, and a lower ratio results in lower variability. Further investigation of *P. ostreatus* is also warranted, with a focus on reducing variability of the ‘optimal’ performing combination, in order to develop an alternative packaging material to Styrofoam with both requisite mechanical properties and consistency.

## Figures and Tables

**Table 1:** Particle size and weight percentage of each fraction in the processed hemp hurd compared to the minimally processed chopped hemp used as a substrate in this study.

Fraction Size (mm)	Hemp Hurd wt %	Chopped Hemp wt %
≥4	40	70
2	40	18
1.5	18	8
<1.5	2	4

**Table 2:** Composition of mycelium-based composites at initial fabrication for each inoculum/substrate ratio for each species.

Fungal Species	Ratio	Inoculum Mass (g)	Substrate Mass (g)	H_2_O (mL)
*Ganoderma sessile*	1:2	750	300	1200
	1:4	375	300	1200
*Trametes versicolor*	1:2	750	300	1200
	1:4	295	236	944
*Pleurotus ostreatus*	1:2	750	300	1200
	1:4	375	300	1200

**Table 3:** MBC mechanical properties with hemp as a substrate, data from the literature review, and results from this study. Fungal species and substrate information, as well as compressive strength (kPa), % deformation, and compressive elastic modulus, are reported.

Fungal Species	Lignocellulosic Substrate	Compressive Strength (kPa)	%Strain	Elastic Modulus (MPa)	Ref.
*P. ostreatus*	100% hemp fiber	18	10	0.2	[43]
*G. lucidum*	wheat straw	70	25		[44]
*T. versicolor*	hemp shives	190 ± 15	10	2.938 ± 0.396	[29]
proprietary	hemp shives	40 ± 10	25		[30]
proprietary (Ecovative)	hemp hurd	124	15	1.138	[45]
*P. ostreatus*	hemp hurd	130 *	10		* estimated from [46]
*Ganoderma* sp.	50% hemp hurd	158.7 ± 12.4	15		[19]
*Ganoderma* sp.	40% hemp hurd	150.9 ± 18.2	15		[19]
*Ganoderma* sp.	30% hemp hurd	128.8 ± 16.0	15		[19]
*G. lucidum*	100% hemp hurd	72 ± 3	10	0.687 ± 0.024	[27]
*T. pubescens*	100% hemp hurd	27 ± 1	10	0.255 ± 0.005	[27]
*G. lucidum*	100% hemp hurd	281 ± 2	35		[27]
*T. pubescens*	100% hemp hurd	104 ± 2	35		[27]
*P. ostreatus*	chopped hemp (1:2)	150.4 ± 29.9	10	1.2 ± 0.1	This study
*G. sessile*	chopped hemp (1:2)	142.1 ± 39	10	1.2 ± 0.3	
*T. versicolor*	chopped hemp (1:2)	159.0 ± 57	10	1.2 ± 0.3	
*P. ostreatus*	chopped hemp (1:4)	194.5 ± 58.5	10	1.7 ± 0.5	
*G. sessile*	chopped hemp (1:4)	169.9 ± 39.6	10	1.3 ± 0.3	
*T. versicolor*	chopped hemp (1:4)	111.4 ± 32	10	0.9 ± 0.3	

## Data Availability

The original data presented in the study are openly available at Mendeley: Bhaskar, Radika (2026), ‘Mycelium based composites physical and mechanical characteristics’, Mendeley Data, V1, doi: 10.17632/zd9ncf85k3.1and upon request.

## Supplementary Materials

The following supporting information can be downloaded at https://www.mdpi.com/article/10.3390/polym18030400/s1. Table S1: Two-way ANOVA results; Figure S1: Interaction effect plots.
