# Supplementary material for: Mycelium-Based Composites Using Minimally Processed Industrial Hemp Biomass: Impact of Species and Feedstock Ratio on Mechanical Performance Compared to Polystyrene Packaging
Source: Polymers (Basel). 2026 Feb 3;18(3):400. doi: 10.3390/polym18030400 (PMC12899536; doi:10.3390/polym18030400)

Supplementary Material

Table S1: Two-way ANOVA table results for each model

Factor	df	SS	F	*p*
Model: Density ~ Species X Ratio				
Species	2	4258.3	3.1	0.052
Ratio	1	16216.0	23.9	**1E-05**
Species x Ratio	2	1216.1	0.89	0.41
Residual	52	35204.5		
Model: Compressive Strength ~ Species X Ratio				
Species	2	15965.5	4.3	0.019
Ratio	1	2014.8	1.1	0.3
Species x Ratio	2	21085.0	5.7	**0.006**
Residual	52	97033.1		
Model: Compressive Modulus ~ Species X Ratio				
Species	2	1.7	8.5	0.0007
Ratio	1	0.2	2.4	0.13
Species x Ratio	2	1.3	6.4	**0.003**
Residual	52	5.1		

Figure S1: Interaction effect plots from 2-way ANOVA analysis

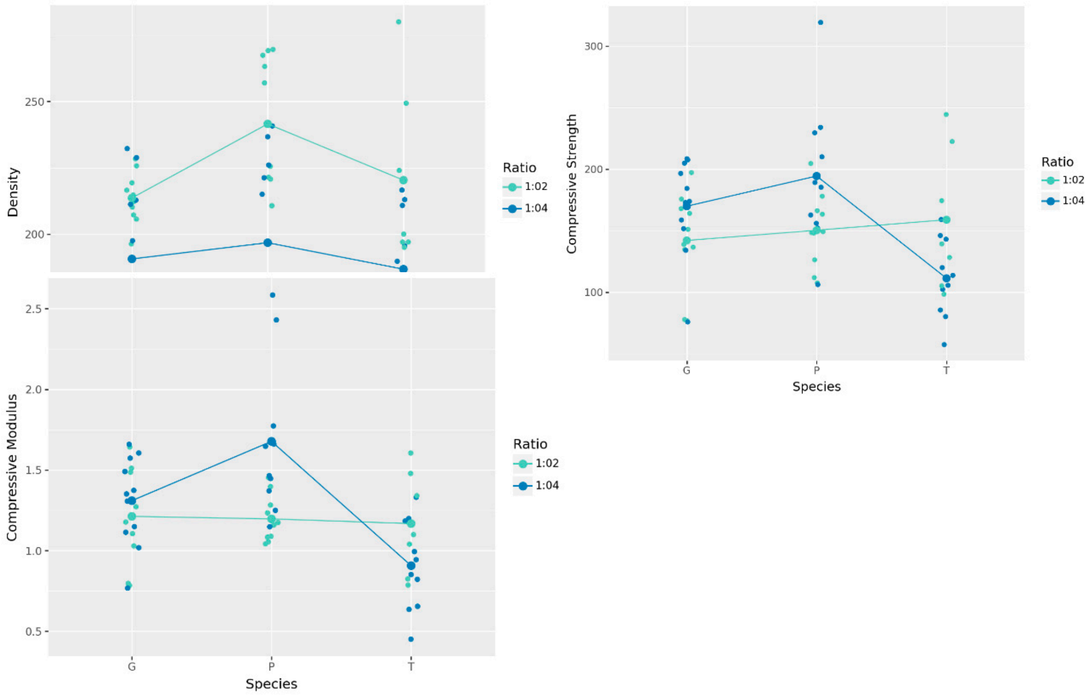

Supplement: Supplementary file 1 [file polymers-18-00400-s001.zip › polymers-4103915-supplementary.pdf]
